# Impact of first and second/third wave of COVID-19 pandemic on post-acute cardiovascular outcomes in Lombardy

**DOI:** 10.3389/fcvm.2023.1244002

**Published:** 2023-09-12

**Authors:** Luisa Ojeda-Fernández, Marta Baviera, Andreana Foresta, Mauro Tettamanti, Antonella Zambon, Giulia Macaluso, Simone Schena, Olivia Leoni, Ida Fortino, Maria Carla Roncaglioni, Gianfranco Parati

**Affiliations:** ^1^ Laboratory of Cardiovascular Prevention, Istituto di Ricerche Farmacologiche Mario Negri IRCCS, Milan, Italy; ^2^ Laboratory of Geriatric Epidemiology, Istituto di Ricerche Farmacologiche Mario Negri IRCCS, Milan, Italy; ^3^ Department of Statistics and Quantitative Methods, University of Milano Bicocca, Milan, Italy; ^4^ Unità Organizzativa Osservatorio Epidemiologico Regionale, Lombardy Region, Milan, Italy; ^5^ Department of Cardiology, Ospedale San Luca, IRCCS Istituto Auxologico Italiano, Milan, Italy; ^6^ Department of Cardiovascular, Neural and Metabolic Sciences, Ospedale San Luca, IRCCS Istituto Auxologico Italiano, Milan, Italy; ^7^ Department of Medicine and Surgery, University of Milano-Bicocca, Milan, Italy

**Keywords:** COVID-19, SARS-CoV-2, post-acute outcomes, cardiovascular outcomes, pandemic waves

## Abstract

**Background:**

COVID-19 has been associated with a higher risk of post-acute complications. Our aim was to analyze and compare post-acute cardiovascular complications of COVID-19 survivors of the first and second/third pandemic waves in Lombardy, in both hospitalized and non-hospitalized COVID-19 patients.

**Methods and results:**

We included adults aged ≥40 years infected during the first and second/third waves of COVID-19 pandemic. The follow-up initiated 30 days after COVID-19 diagnosis and continued up to 9 months. Hazard ratios (HRs) and 95% confidence intervals (CIs) of the post-acute cardiovascular outcomes were calculated against an inverse probability treatment weighted control group. Subgroup analysis were performed by age classes, sex, previous cardiovascular disease and stratified by COVID-19 hospitalization status to explore the impact of COVID-19 severity on outcomes. Compared to the control group, COVID-19 patients had an increased risk of hospitalization for any cardiovascular complications (HR 1st wave 1.53 95% CI: 1.38–1.69; HR 2nd/3rd wave 1.25 95% CI: 1.19–1.31) and for individual cardiovascular outcomes, although HRs were higher in COVID-19 group from the 1st pandemic wave. The results were confirmed in the subgroup analyses. Of note, the risk for any cardiovascular disease was also evident even among individuals who were not hospitalized during the acute phase of the infection.

**Conclusion:**

Our results provide evidence that COVID-19 is a risk factor for post-acute cardiovascular complications among different pandemic waves regardless of COVID-19 severity, age, sex and a history of cardiovascular diseases. Care strategies of people with COVID-19 should include cardiac monitoring.

## Graphical Abstract

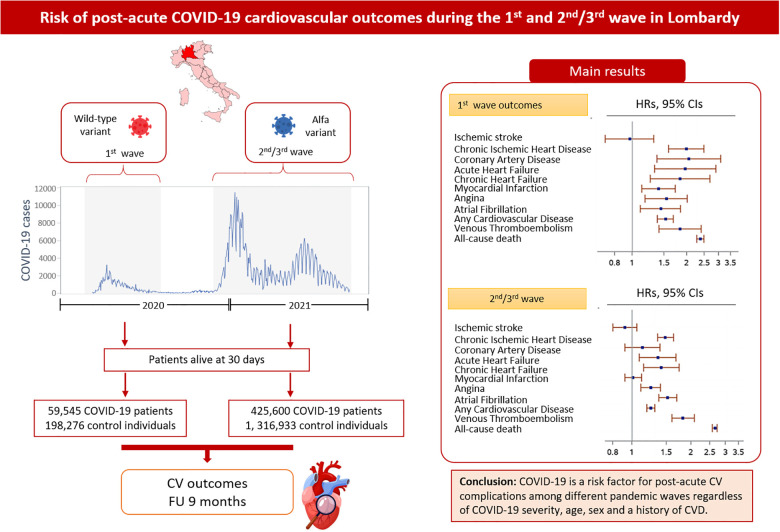

## Introduction

COVID-19 pandemic, which is caused by SARS-CoV-2, spread globally and caused mortality at an unprecedented scale (1). Although acute respiratory distress syndrome was one of the hallmarks of the COVID-19 viral infection, multi-organ system complications were observed in patients with severe disease (2). In particular, COVID-19 patients could present cardiovascular (CV) disorders, such as myocardial injury, arrhythmias, acute coronary syndrome (ACS) and venous thromboembolism (VTE) (3–6)*.* In addition, a wide range of CV complications have been also reported after the acute phase supporting an interplay between SARS-CoV-2 and CV system (7–9). The reason is unclear but may be related directly to virally mediated vascular endothelial injury or indirectly to the inflammatory process accompanying the immune response (10–13). Long-term CV sequelae in COVID-19 patients are currently under investigation worldwide. So far, most published observational studies have mainly focused on patients admitted to hospital for COVID-19 (14–16), or analyzed the general population but with a short/mid-term post-infection follow-up (17–19). To our knowledge, only two large population based studies have analyzed the incidence of long-term CV outcomes (20, 21). However, these studies have pooled data from different COVID-19 pandemic waves, although the disease severity and the clinical characteristics were driven by different variants of the coronavirus (22, 23). Of note, in Lombardy, one of the first regions to experience a rapid increase in numbers of COVID-19 infections and related deaths (24), the first wave of COVID-19 pandemic overwhelmed the capacity of hospitals, particularly intensive care units, and had a stronger impact on health of people compared to the second wave of the pandemic (23, 25).

With the ongoing COVID-19 pandemic and the increasing number of patients recovered from the disease, growing evidence is needed about CV long-term effects to improve care strategies in COVID-19 survivors. We investigated the prevalence of post-acute CV outcomes in a large sample of hospitalized and non-hospitalized COVID-19 patients in comparison to a matched control group in the 1st and 2nd/3rd waves of the pandemic in Lombardy.

## Methods

### Data source

Our study used two linkable administrative databases of the of the Lombardy region. One of them was a healthcare utilization database aimed to facilitate Regional Health Service (RHS) management and includes information on demographic data, drug prescriptions and hospital records of Lombardy residents. The second administrative database was the *Database DB Covid-19* that was the registry of patients with a confirmed diagnosis of SARS-CoV-2 infection, with the aim of monitoring ascertained infections of SARS-CoV-2 and hospital admissions, emergency room accesses, and deaths due to COVID-19. *Database DB Covid-19* was used to identify COVID-19 patients and RHS database to identify control individuals. A unique identification code allows linkage of all databases. For more details, see Supplementary Material.

Healthcare in Italy is publicly funded for all residents, irrespective of social class or employment, and every resident is assigned a personal identification number kept in the National Civil Registration System. All residents are assisted by general practitioners (GPs) under the NHS. To ensure privacy, each identification code was automatically de-identified. The inverse process being allowed only to the RHA on request from judicial authorities. According to Italian law, studies using retrospective aggregated data from administrative databases that do not involve direct access by investigators to individual patients' data, do not require approval or notification from an Ethics Committee/IRB or patients' informed consent.

### Study population and exposure

**Figure 1:**
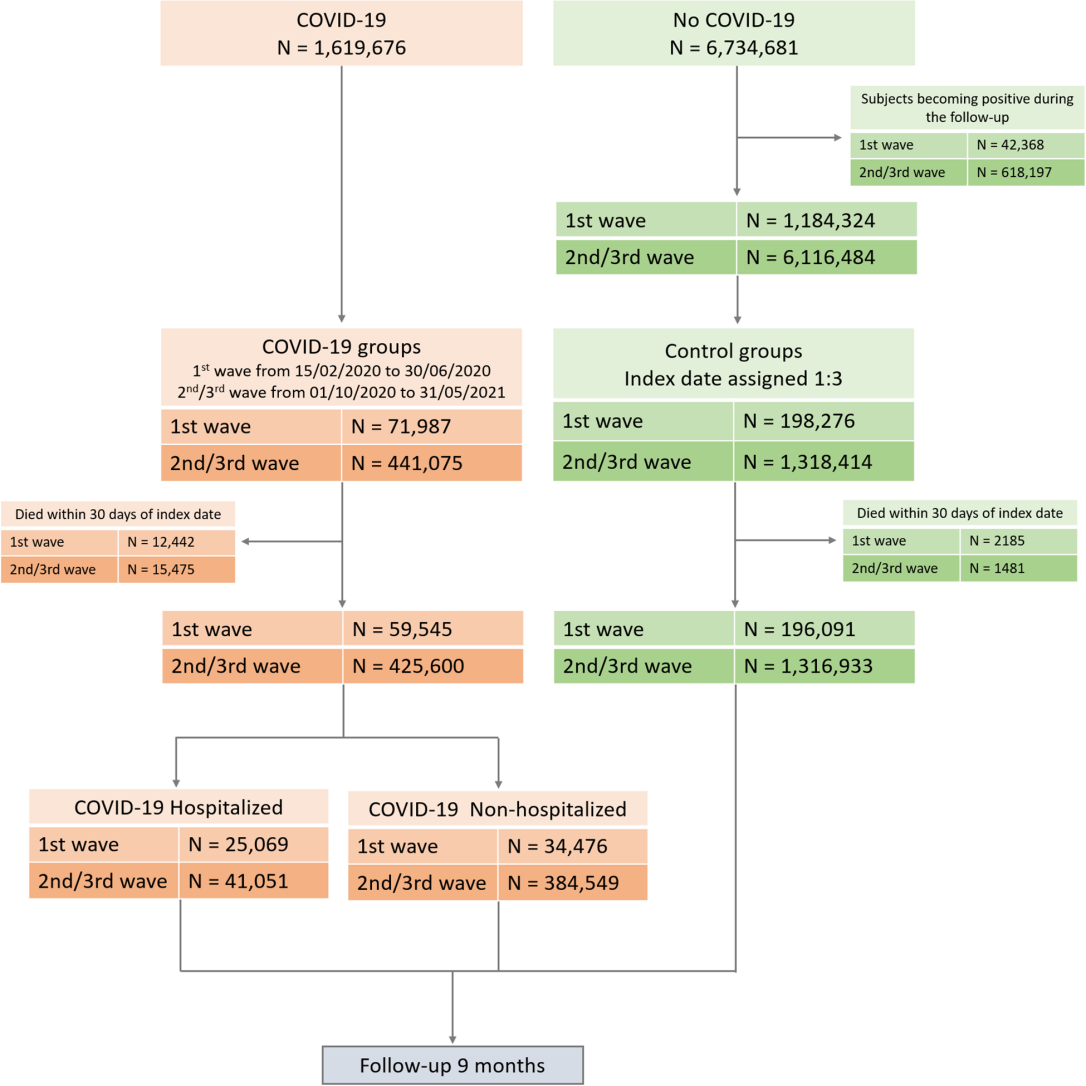
Flow-chart of cohort construction.

We selected patients with a diagnosis of COVID-19 aged 40 years and older from 15 February to 30 June 2020 (1st wave) and from 1 October 2020 to 31 May 2021 (2nd/3rd wave). In Italy, there was not a strict separation between the 2nd and 3rd wave of COVID-19 pandemic, thus in the present study they are considered together. Index date is defined as the date of positive test for SARS-CoV-2 or hospital admission for COVID-19, whichever comes before. For each COVID-19 patient, up to three control individuals were randomly assigned and available in the control dataset, with the same distribution of age (±3 years), sex and GP. We excluded control individuals with a COVID-19 diagnosis during the follow-up. To examine post-acute CV outcomes, we then selected individuals from the COVID-19 group who were alive 30 days after the index date. For further analysis, the COVID-19 cohort was also divided by non-hospitalized or hospitalized patients during the acute phase of COVID-19. Also in the control group, subjects alive 30 days after the assigned index date were analyzed (Figure 1).

### Study variables

Demographic data were recorded at the time of inclusion. History of comorbidities was collected in the four years before the index date using hospital records as primary diagnosis and up to five co-existing conditions. The Charlson comorbidity index (26) as a proxy of comorbidities was also retrieved. Exposures to medications of interest were traced in the 12 months before index date electrocardiogram (ECG) and cardiology visits were also collected in the previous 12 months before index date (Supplementary Material, ATC and ICD-9 codes).

### Outcomes and follow-up

Follow-up observation started 30 days after the index date and proceeded up until the minimum date between the date of death or the date of the event or the end of the study period (9 months after entering the cohorts). We analyzed the following CV outcomes: ischemic stroke, chronic ischemic heart disease, coronary artery disease, acute heart failure, chronic heart failure, myocardial infarction, angina and atrial fibrillation using hospital records as primary diagnosis. We also analyzed a composite CV outcome defined as any CV disease that consisted of at least one of the CV outcomes mentioned above. In addition, we reported the frequency of hospitalizations due to venous thromboembolism (VTE) and all-cause death. Finally, we evaluated the number cardiology visits, ECG and emergency room access during the follow-up. To test the specificity of any association between COVID-19 and CV outcomes, we also included a negative control composite outcome.

### Statistical analysis

Descriptive analysis was performed to describe the cohort baseline characteristics: COVID-19 and control individuals. Continuous variables were reported as mean and standard deviation (SD) and dichotomous variables as frequencies and percentages. Baseline continuous variables were compared using a *t*-test, and categorical variables were compared with a *χ*^2^ test.

Using a Cox proportional hazards model, we estimated hazard ratios (HRs) and 95% confidence intervals (CIs) to analyze the association between COVID-19 and risk of CV outcomes of interest and all-cause death. HRs were computed taking advantage of Inverse Probability Treatment Weighting (IPTW). We used IPTW to minimize the repercussions of selection bias (27). The average of the inverse predicted probabilities must be approximately equal to 1 to consider the well-behaved weights that led to a small variance of the effect estimate (28). HRs were adjusted for sex, age, pre-existing condition, previous ECG and cardiology visits, and medications of interest.

HRs for any CV disease and all-cause death between COVID-19 and control cohorts were estimated in subgroup analyses by sex (M/F), age (40–65 vs. >65 years) and previous CV disease (yes/no). Additional HRs were also calculated according to COVID-19 acute phase care setting (hospitalized and non-hospitalized patients) in comparison with control cohort.

A *P*-value <0.05 was used for statistical significance. All analyses were done using SAS version 9.4 (SAS Institute).

## Results

Figure 1 shows the flow-chart of the study. A total of 59,545 COVID-19 patients and 196,091 control individuals, and 425,600 COVID-19 patients and 1,316,933 control individuals, for the 1st and the 2nd/3rd wave respectively, were included in the analysis. Baseline characteristics of COVID-19 survivors and control groups for 1st and 2nd/3rd waves of the pandemic are reported in Table 1. Survivors to the COVID-19 acute phase during 1st and 2nd/3rd waves were slightly younger and had more pre-existing conditions compared to control individuals. COVID-19 patients in the 2nd/3rd wave were younger and with less pre-existing conditions than COVID-19 patients in the 1st wave. Baseline characteristics of non-hospitalized and hospitalized COVID-19 patients vs. control individuals are reported in Supplementary Tables S1, S2. In general, non-hospitalized COVID-19 patients were younger, more likely to be females and less exposed to medication compare to control group, while hospitalized COVID-19 patients were older, more likely to be men with more pre-existing clinical conditions and received more medications compared to the control group. The largest difference in pre-existing clinical conditions prevalence between both COVID-19 groups (non-hospitalized and hospitalized) and the control group was observed for chronic obstructive disease. The mean follow-up in the COVID-19 and control groups was 222 ± 46 and 230 ± 30 days for the 1st wave, and 237 ± 29 and 240 ± 16 days for 2nd/3rd wave, respectively.

**Table 1:** Baseline characteristics of COVID-19 patients during the 1st and 2nd/3rd wave of the pandemic and randomly assigned control subjects alive after 30 days, before inverse probability weighting.

Variables	1st wave			2nd/3rd wave		
	COVID-19 *N* = 59,545	Control *N* = 196,091	*P*-value	COVID-19 *N* = 425,600	Control *N* = 1,316,933	*P*-value
Age (years), mean ± SD	64.97 ± 14.97	66.65 ± 14.73	<0.0001	59.26 ± 13.48	60.12 ± 13.87	<0.0001
Age groups (years), *n* (%)						
40–59	25,694 (43.15)	73,638 (37.55)	<0.0001	249,648 (58.66)	745,308 (56.59)	<0.0001
60–79	20,920 (35.13)	73,782 (37.63)		131,267 (30.84)	408,585 (31.03)	
≥80	12,931 (21.72)	48,671 (24.82)		44,685 (10.50)	163,040 (12.38)	
Gender (female)	32,035 (53.80)	97,938 (49.95)	<0.0001	221,581 (52.06)	681,932 (51.78)	0.0014
Pre-existing clinical conditions, *n* (%) (in the previous 4 years)						
Ischemic stroke	754 (1.27)	1,400 (0.71)	<0.0001	1,878 (0.44)	4,160 (0.32)	<0.0001
Acute heart failure	251 (0.42)	498 (0.25)	<0.0001	677 (0.16)	1,313 (0.10)	<0.0001
Chronic heart failure	403 (0.68)	747 (0.38)	<0.0001	1,105 (0.26)	2,047 (0.16)	<0.0001
Myocardial infarction	988 (1.66)	2,271 (1.16)	<0.0001	3,713 (0.87)	8,578 (0.65)	<0.0001
Chronic ischemic heart disease	2,090 (3.51)	4,595 (2.34)	<0.0001	6,794 (1.60)	15,241 (1.16)	<0.0001
Coronary artery disease	219 (0.37)	547 (0.28)	0.0005	987 (0.23)	2,161 (0.16)	<0.0001
Angina	637 (1.07)	1,652 (0.84)	<0.0001	2,691 (0.63)	6,446 (0.49)	<0.0001
Cardiogenic shock	59 (0.10)	53 (0.03)	<0.0001	149 (0.04)	258 (0.02)	<0.0001
Atrial fibrillation	2,623 (4.41)	4,957 (2.53)	<0.0001	7,205 (1.69)	14,983 (1.14)	<0.0001
Venous thromboembolism	1,031 (1.73)	1,030 (0.53)	<0.0001	2,309 (0.54)	3,542 (0.27)	<0.0001
Chronic kidney disease	1,370 (2.30)	1,871 (0.95)	<0.0001	3,169 (0.74)	5,383 (0.41)	<0.0001
Dialysis	271 (0.46)	142 (0.07)	<0.0001	485 (0.11)	473 (0.04)	<0.0001
Chronic obstructive disease	7,447 (12.51)	3,611 (1.84)	<0.0001	10,068 (2.37)	11,215 (0.85)	<0.0001
Asthma	216 (0.36)	259 (0.13)	<0.0001	576 (0.14)	1,074 (0.08)	<0.0001
Liver disease	632 (1.06)	1,003 (0.51)	<0.0001	1,804 (0.42)	4,036 (0.31)	<0.0001
Charlson index (years), mean ± SD (in the previous 12 months)	0.19 ± 0.87	0.06 ± 0.39	<0.0001	0.06 ± 0.42	0.03 ± 0.26	<0.0001
CV healthcare utilization markers, *n* (%) (in the previous 12 months)						
ECG	9,818 (16.49)	31,617 (16.12)	0.0344	43,588 (10.24)	116,390 (8.84)	<0.0001
Cardiology visit	7,494 (12.59)	24,863 (12.68)	0.5462	31,765 (7.46)	87,789 (6.67)	<0.0001
Medications, *n* (%) (in the previous 12 months)						
Antidiabetic drugs	6,064 (10.18)	17,808 (9.08)	<0.0001	30,063 (7.06)	80,909 (6.14)	<0.0001
ACE-I/ARBS	18,462 (31.01)	64,995 (33.15)	<0.0001	104,768 (24.62)	305,393 (23.19)	<0.0001
Beta blockers	11,400 (19.15)	37,350 (19.05)	0.5943	59,603 (14.00)	173,023 (13.14)	<0.0001
Diuretics	6,244 (10.49)	17,726 (9.04)	<0.0001	24,382 (5.73)	68,160 (5.18)	<0.0001
Ca-antagonists	6,714 (11.28)	22,984 (11.72)	0.0030	33,629 (7.90)	98,089 (7.45)	<0.0001
Lipid lowering drugs	11,255 (18.90)	40,497 (20.65)	<0.0001	64,226 (15.09)	188,857 (14.34)	<0.0001
Antiplatelet drugs	7,843 (13.17)	25,901 (13.21)	0.8147	34,457 (8.10)	100,857 (7.66)	<0.0001
Oral anticoagulant drugs	3,396 (5.70)	10,989 (5.60)	0.3575	14,714 (3.46)	42,837 (3.25)	<0.0001
Heparin	3,395 (5.70)	9,286 (4.74)	<0.0001	33,881 (7.96)	41,620 (3.16)	<0.0001
Drugs for respiratory disease	6,400 (10.75)	18,835 (9.61)	<0.0001	35,722 (8.39)	81,527 (6.19)	<0.0001
Steroidal anti-inflammatory drugs	5,484 (9.21)	15,101 (7.70)	<0.0001	50,871 (11.95)	71,708 (5.45)	<0.0001
No-steroidal anti-inflammatory drugs	8,286 (13.92)	27,242 (13.89)	0.8870	53,408 (12.55)	133,312 (10.12)	<0.0001

ACE-I, angiotensin-converting enzyme inhibitors; ARBs, angiotensin II receptor agonist blockers; IQR, interquartile range; SD, standard deviation.

**Figure 2:**
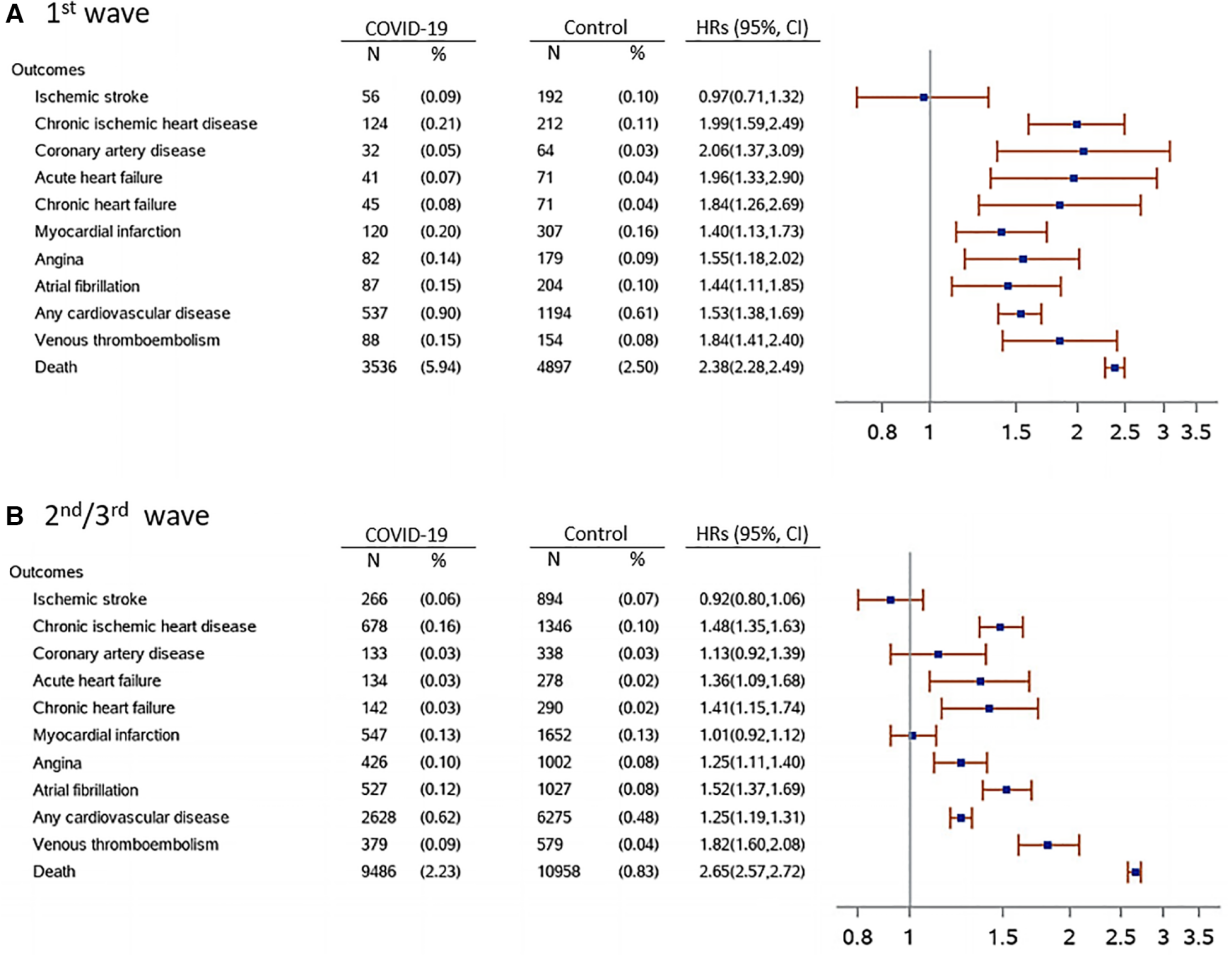
Post-acute COVID-19 cardiovascular outcomes during the 1st and 2nd/3rd wave compared with the control cohort. Outcomes were ascertained 30 days after the COVID-19 diagnosis until the end of follow-up (up to 9 months after the index date). Any cardiovascular disease composite outcome consisted of hospitalization by ischemic stroke, chronic ischemic heart disease, coronary artery disease, acute heart failure, chronic heart failure, myocardial infarction, angina and atrial fibrillation. Hazard ratios (HRs) were computed after IPTW. HRs (95% CI) were adjusted for sex, age, pre-existing condition, medications of interest and previous cardiology visits and ECG. IPTW 1st wave (mean, median [q1, q3]) = 1.00, 0.98 [0.93, 1.04]. IPTW 2nd/3rd wave (mean, median [q1, q3]) = 1.00, 0.99 (0.96, 1.02).

After IPTW the average inverse predicted probabilities were around 1 for all groups in comparison with their counterparts, suggesting that covariates were well balanced after inverse probability weighting (28). Absolute standardized difference between COVID-19 and control group before and after IPTW are shown in Supplementary Figures S2, S3. Number of events and adjusted HRs (95% CI) are shown in Figure 2. Significantly higher risks were observed in COVID-19 patients compared with control group for all considered outcomes, except for ischemic stroke in both waves, although differences for coronary artery disease and myocardial infarction were not significant in the 2nd/3rd wave. In both waves the risk of VTE was statistically significant higher in COVID-19 patients compared to controls. A significantly higher number of ECGs, cardiology visits, emergency room accesses and CV re-hospitalizations was reported for COVID-19 patients compared to control group, while differences for CV re-hospitalizations were not statistically significant in COVID-19 patients in the 2nd/3rd wave of the pandemic (Supplementary Tables S3). No significant association was observed between COVID-19 and a composite negative-outcome where an association is not expected (Supplementary Tables S4).

**Figure 3:**
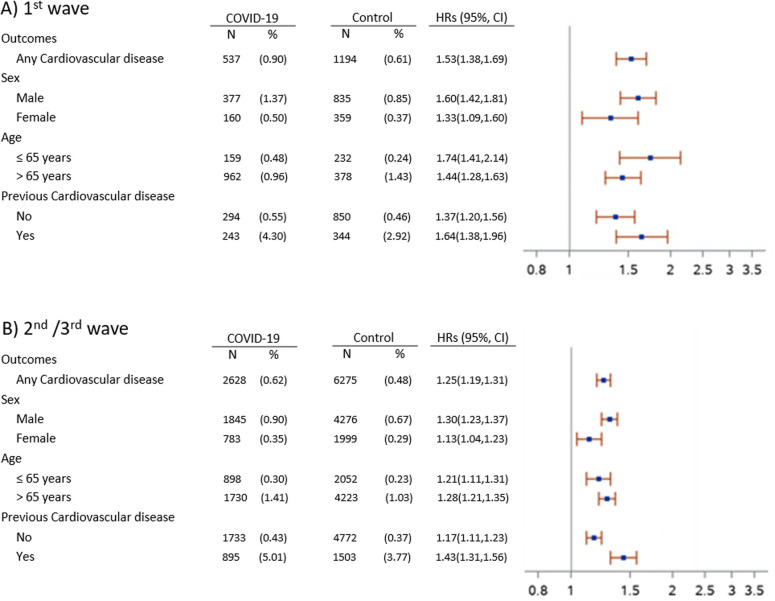
Subgroup analysis of any cardiovascular disease in COVID-19 group compared to control during the 1st (**A**) and 2nd/3rd (**B**) wave of pandemic. Outcomes were ascertained 30 days after the COVID-19 diagnosis until the end of follow-up (up to 9 months after the index date). Any cardiovascular disease composite outcome consisted of hospitalization by ischemic stroke, chronic ischemic heart disease, coronary artery disease, acute heart failure, chronic heart failure, myocardial infarction, angina and atrial fibrillation. Hazard ratios (HRs) were computed after IPTW. HRs (95% CI) were adjusted for sex, age, pre-existing condition, medications of interest and previous cardiology visits and ECG. IPTW 1st wave (mean, median [q1, q3]) = 1.00, 0.98 [0.93, 1.04]. IPTW 2nd/3rd wave (mean, median [q1, q3]) = 1.00, 0.99 (0.96, 1.02).

A significantly higher risk of being hospitalized for any CV disease was seen in COVID-19 patients across all the considered subgroups stratified by sex, age and hospitalization for previous CV disease compared to the control group (Figure 3). A higher risk for any CV disease was also observed in non-hospitalized and hospitalized COVID-19 patients, considered separately, in comparison to the control group, although this difference did not reach the statistically significance for non-hospitalized COVID-19 patients of the 1st wave (Table 2).

**Table 2:** Number of events and HRs (95% CI) for post-acute any cardiovascular disease outcome in hospitalized and non-hospitalized COVID-19 patients compared with the control group during the 1st and 2nd/3rd wave.

Subgroup	1st wave			2nd/3rd wave		
	*N*	(%)	HR (95%, CI)	*N*	(%)	HR (95%, CI)
Control	1,194/196,091	0.61	Referent	6,275/1,316,933	0.48	Referent
Non-hospitalized Covid-19	174/34,476	0.50	1.16 (0.99, 1.36)	1,904/384,549	0.50	1.13 (1.08, 1.19)
Hospitalized Covid-19	363/25,069	1.45	1.84 (1.63, 2.08)	724/41,051	1.76	1.75 (1.61, 1.90)

Outcomes were ascertained 30 days after the COVID-19 diagnosis until the end of follow-up (up to 9 months after the index date). Any cardiovascular disease composite outcome consisted of hospitalization by ischemic stroke, chronic ischemic heart disease, coronary artery disease, acute heart failure, chronic heart failure, myocardial infarction, angina and atrial fibrillation. Hazard ratios (HRs) were computed after IPTW. HRs (95% CI) were adjusted for sex, age, pre-existing condition, medications of interest and previous cardiology visits and ECG. IPTW 1st wave [mean, median (q1, q3)] = 1.00, 0.98 [0.93, 1.04]. IPTW 2nd/3rd wave [mean, median (q1, q3)] = 1.00, 0.99 (0.96, 1.02).

## Discussion

This population-based study evaluated the post-acute CV outcomes among 59,545 COVID-19 cases from the 1st wave and 425,600 COVID-19 cases from the 2nd/3rd wave of the COVID-19 pandemic in Lombardy. To our knowledge, this is the largest study analyzing the prevalence of post-acute CV complications among different COVID-19 outbreaks, where Wuhan original strain and Alfa variant were predominant in Italy during the 1st and 2nd/3rd waves, respectively (29). With the exception of ischemic stroke, we found an increased risk of post-acute CV outcomes in COVID-19 patients compared to control individuals up to 9 months of follow-up in both waves. The risk of any CV disease remained higher across all subgroups of patients stratified by sex, age and hospitalizations for previous CV disease. In comparison with control individuals, the risk of any CV disease in the post-acute phase was higher also in non-hospitalized COVID-19 patients, although the HRs were lower than those seen in hospitalized COVID-19 patients. In addition, a higher frequency of cardiology visits and ECGs during the observation period in both waves was reported for COVID-19 patients, suggesting that these patients were more likely to be monitored for CV symptoms or CV complications than control group after the acute phase.

In general, we observed a higher risk of having CV complications in COVID-19 patients infected during the 1st pandemic wave. The differences observed may be related to differences in COVID-19 population between waves: COVID-19 patients infected during the 1st wave were older and with a higher prevalence of CV diseases compared to COVID-19 patients infected during the 2nd/3rd wave. Moreover, after the 1st wave of pandemic, significant changes have been made in the health care system increasing the total number of ICU beds and involving the primary care in the initial management of COVID-19 (30). This may has positively impacted on patient's outcomes. In addition, improvements in COVID-19 treatment might have driven the lower prevalence of post-acute CV outcomes that we have observed in patients infected during the 2nd/3rd wave. This may be due to the use of the corticosteroid dexamethasone that, beyond its efficacy to reduce mortality in patients who were hospitalized for COVID-19 (31, 32), showed to decrease cardiac inflammation in patients with myocarditis (33). This hypothesis is also in line with Wu et al. (34) that reported a markedly decrease of cardiac inflammation, cardiomyocyte injury and microvascular thrombogenicity in fatal COVID-19 cases in second wave compared to first wave patients. Of note, cardiac abnormalities diagnosed by transthoracic echocardiography seem to alleviate gradually after the acute phase but their evolution is associated to patients' age, the severity of the acute infection but also with the pathogeny of different viral strains (35).

Cardiac damage during the acute phase of COVID-19 is hypothesized to be caused by direct and indirect cardiac injury. Direct cardiac injury is driven by SARS-CoV-2 cardiomyocytes infiltration that highly express ACE2 receptor. Indirect injury could be mediated by respiratory failure induced hypoxic injury, overwhelming cytokine release, systemic inflammation, hypercoagulability, Renin-Angiotensin-Aldosterone System dysregulation (RAAS), plaque destabilization, and myocardial supply–demand mismatch (13). COVID-19 is potentially the cause of a wide range of post-acute CV complications. However, the underlying mechanisms for CV sequelae are not entirely understood and it has been hypothesized that altered physiological states and mechanical/anatomical damage to the cardiac system during the acute phase of COVID-19 were involved (13). These mechanisms include persistent hyperactivated immune response and the persistence of the virus in different organs or tissues that may trigger post-inflammation residual damage, immune system dysregulation and inadequate antibody response (12, 13, 36). Moreover, integration of the SARS-CoV-2 genome into DNA of infected human cells has been hypothesized as a mechanism for continued activation of the immune-inflammatory-pro-coagulant cascade (37, 38). Besides biological mechanisms mentioned before, changes in daily life imposed by restrictive measures to contain the spread of SARS-CoV-2, have been associated to unhealthy lifestyle habits leading to an increase in CV risk during COVID-19 pandemic, as recently reported by Solfanelli et al. in a population of healthcare workers (39).

### Comparison with other studies

The prevalence of post-acute CV sequelae has varied across and within various studies. This may reflect differences in methodology, in the study populations and care setting during the acute phase, pandemic waves, burden of co-morbidities and/or length of follow-up (10, 40–42). A prospective cohort study conducted in Italy including only COVID-19 patients during the 1st pandemic wave reported that female sex, in-hospital acute heart failure and atrial fibrillation were predictors of major CV complications (16).

Few matched control studies have reported short/middle-term CV outcomes with some controversial results. Daugherty et al. (17) observed an increased risk for DVT, PE and stroke in the 4 months post-infection during the 1st pandemic wave in the USA. Raisi-Estabragh et al. (19) reported an increased risk of all CV outcomes considered (MI, stroke, heart failure and VTE) in the 5 months following infection only for COVID-19 hospitalized patients from the UK Biobank registry, while non-hospitalized patients had only an increased risk of VTE. Our results are in line with those coming from matched cohort studies conducted in COVID-19 survivors in the USA reporting an increased risk of long-term CV outcomes of COVID-19 among patients in the US Veterans Health Administration (VHA) system (20) and in the TriNetX US collaborative network (21). These studies did not differentiate between the two pandemic waves and were focused on patients without pre-existing CV conditions although is known that pre-existing CV condition is associated with more severe illness (16, 43, 44).

### Strengths and limitations

Our study has several strengths. First we analyzed the whole population of COVID-19 cases of Lombardy, the first region in Western world to experience a rapid increase in number of COVID-19 cases. Second, we analyzed post-acute CV outcomes differentiating between COVID-19 waves as the impact of COVID-19 pandemic in healthcare system and demographic and clinical characteristics of COVID-19 patients were extremely different within the 1st and the 2nd/3rd wave of the pandemic in Lombardy. Furthermore, administrative databases are recognized as a reliable tool to analyze outcomes of large patient cohorts that represent real clinical care, since data are collected over time in a standardized way and at low cost (45, 46) and enabled us to conduct a matched analysis of post-acute outcomes. Finally, we used validated and standardized outcome definitions and adjusted the analyses for a large set of available variables such as age, sex, pre-existing clinical conditions and medications as well as for ECGs and cardiology visits before COVID-19 diagnosis.

This study suffers also from some known limitations characteristic of the administrative databases. Although we adjusted for a set of pre-defined variables and HRs were also computed after IPTW to achieve a balanced distribution of confounders across treatment groups, specific information such as body mass index, smoke, glycaemia, eGFR (estimated glomerular filtration rate), etc. were not available in our database and so we cannot rule out confounding by indications. We couldn't assess the death for CV reasons because this information was not available. Additionally, subjects under 40 years were not included in our analysis since we have access to data of residents 40 years or older according to EPIFARM-Pharmaco-epidemiology Agreement between the Regional Health Service of the Lombardy Region and Istituto di Ricerche Farmacologiche Mario Negri IRCCS. It is possible that some people, in spite of having had COVID-19, might have been included in the control group because were not tested for SARS-CoV-2 infection. Finally, we did not exclude individuals vaccinated for COVID-19, but it should be considered that the vaccination campaign in Italy effectively began in January 2021. By 15 May 2021 only 13.1% of citizens completed the COVID-19 vaccination in Lombardy (47), thus we assume that, because of such a low prevalence, the inclusion of vaccinated individuals did not affect the reliability of our analysis.

## Conclusions

In conclusion, in this large population-based study, we found that patients infected during the 1st and 2nd/3rd waves of the COVID-19 pandemic had a higher risk of post-acute CV complications compared to a control group. We also found that the risks were lower for individuals infected during the 2nd/3rd wave compared to those infected during the 1st wave. With the ongoing pandemic and millions of patients infected worldwide, the number of survivors with potential CV sequelae will continue to grow. Thus, future studies are needed to address post-acute CV complications in different populations, taken into account SARS-CoV-2 variants, re-infection, vaccination status and longer follow-ups. Our results strongly suggest that care strategies for people with a history of COVID-19 should also include cardiac monitoring regardless of COVID-19 severity.

All authors take responsibility for all aspects of the reliability and freedom from bias of the data presented and their discussed interpretation.

## Data Availability

The datasets presented in this article are not readily available because the data that support the findings of this study are available from Lombardy Region, but restrictions apply to the availability of these data, which were used under license for the current study, and so are not publicly available. Data are however available from the Lombardy Region upon reasonable request. Requests to access the datasets should be directed to https://www.regione.lombardia.it.
