# Supplementary material for: Impact of first and second/third wave of COVID-19 pandemic on post-acute cardiovascular outcomes in Lombardy
Source: Front Cardiovasc Med. 2023 Sep 12;10:1244002. doi: 10.3389/fcvm.2023.1244002 (PMC10536134; doi:10.3389/fcvm.2023.1244002)
Supplement: Supplementary file 1 [file Datasheet1.docx]

**SUPPLEMENTARY MATERIAL**

**Impact of first and second/third wave of COVID-19 pandemic on post-acute cardiovascular outcomes in Lombardy.**

Luisa Ojeda-Fernández, Marta Baviera, Andreana Foresta, Mauro Tettamanti, Antonella Zambon, Giulia Macaluso, Simone Schena, Olivia Leoni, Ida Fortino, Carla Roncaglioni, Gianfranco Parati

**Figure S1.** Daily confirmed COVID-19 cases in Lombardy region.

**
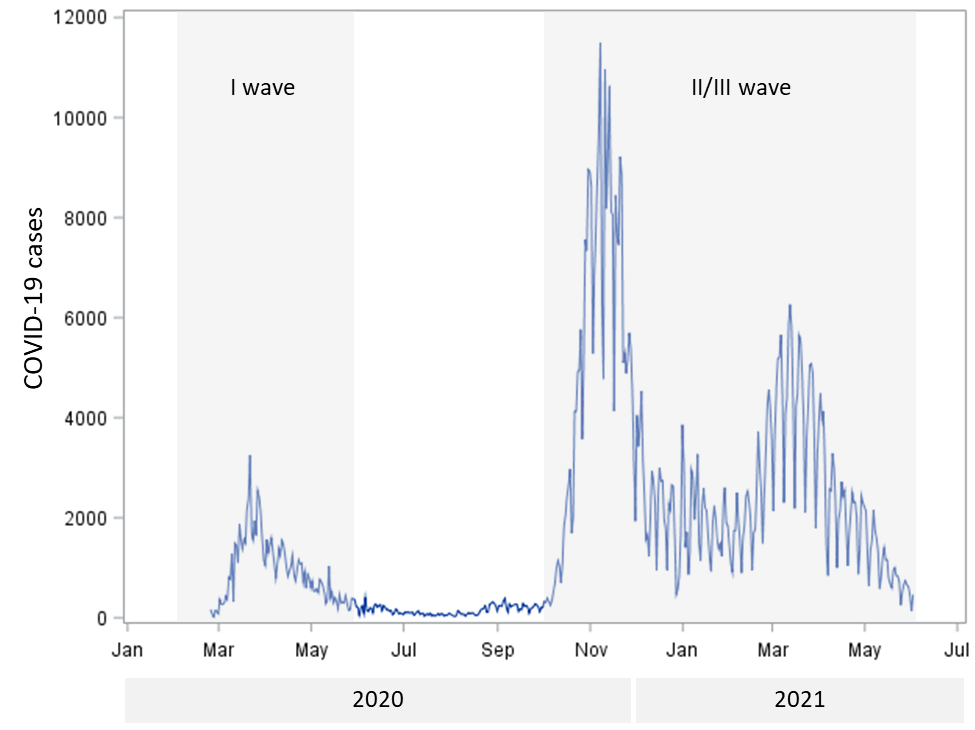
**

Source: https://opendatadpc.maps.arcgis.com/apps/dashboards/b0c68bce2cce478eaac82fe38d4138b1

# **Data source**

Our study used two linkable administrative databases of the of the Lombardy region. One database is the healthcare utilization database aimed to facilitate Regional Health Service (RHS) management and includes information on demographic data, drug prescriptions and hospital records of Lombardy residents. These data were available for subjects 40 years or older. The drug prescription database contains the medication name and anatomic therapeutic chemical (ATC) classification code, quantity, and date of dispensation of drugs reimbursed by the National Health Service (NHS). No information is available on drugs dispensed in hospital. The hospital database contains information on the date of admission, discharge, death, primary diagnosis, and up to five co-existing clinical conditions and procedures received. The diagnoses, uniformly coded according to the 9th International Code of Diseases (ICD-9-CM) and standardized in all Italian hospitals, are compiled by the hospital specialists directly in charge of the patients and are validated by hospitals against detailed clinical-instrumental data, as they determine reimbursement from the NHS. Drug prescriptions and hospital records are available from 2000 to 2021. The second administrative database is the Database DB Covid-19 that is the registry of patients with a confirmed diagnosis of SARS-CoV-2 infection, which was established since 21 February 2020 (i.e., on the date of the first ascertained diagnosis in Lombardy) with the aim of monitoring ascertained infections of SARS-CoV-2 and hospital admissions, emergency room accesses, and deaths due to COVID-19. Diagnoses of COVID-19 were revealed to the Regional Health Authority (RHA) from the following several sources: public and private hospitals, general practitioners, municipal registries, and laboratories accredited by the RHA. A unique identification code allows linkage of all databases.

Access to data is allowed within Agreement between the Istituto di Ricerche Farmacologiche Mario Negri and the Lombardy Healthcare System. Healthcare in Italy is publicly funded for all residents, irrespective of social class or employment, and every resident is assigned a personal identification number kept in the National Civil Registration System. All residents are assisted by general practitioners (GPs) under the NHS. To ensure privacy, each identification code was automatically de-identified. The inverse process being allowed only to the RHA on request from judicial authorities. According to Italian law, studies using retrospective aggregated data from administrative databases that do not involve direct access by investigators to individual patients’ data, do not require approval or notification from an Ethics Committee/IRB or patients’ informed consent.

**Figure S2.** Standardized mean differences before and after IPTW between COVID-19 and control groups of the 1^st^ pandemic wave.


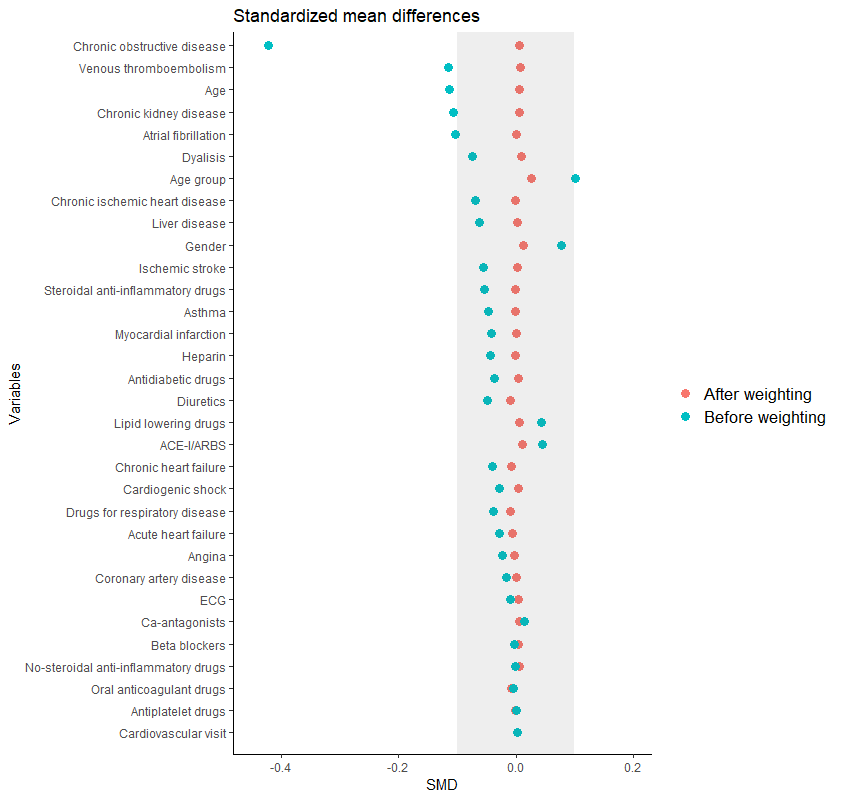


**Figure S3.** Standardized mean differences before and after IPTW between COVID-19 and control groups of the 2^nd^/3^rd^ pandemic wave.


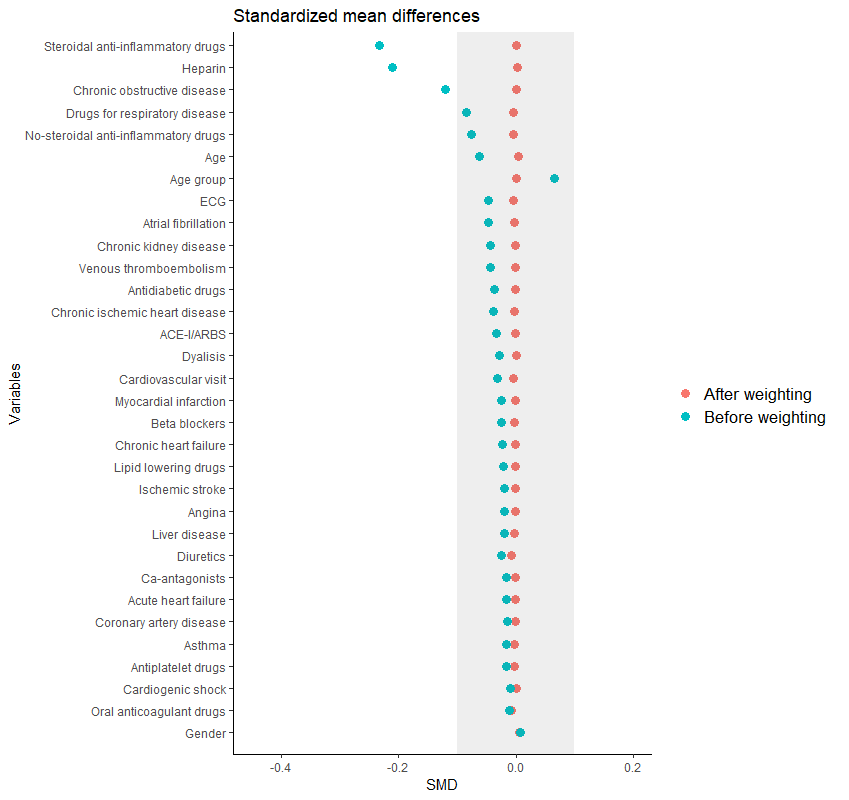


**Table S1.** Baseline characteristics of COVID-19 hospitalized and non-hospitalized patients and control subjects alive after 30 days, before IPTW, during the 1^st^ wave of the pandemic.

| **Variables** | **Control**  **N=196091** | **Non-hospitalized**  **N=34476** | **P-value**  **vs Ctrl** | **Hospitalized**  **N=25069** | **P-value**  **vs Ctrl** |
| --- | --- | --- | --- | --- | --- |
| **Age** (ys)**,** mean ± SD | 66.87 ± 14.82 | 62.84 ± 15.98 | <0.0001 | 67.90 ± 12.90 | <0.0001 |
| **Age groups** (ys), n (%) | | | | | |
| 40-59 | 73638 (37.55) | 18416 (53.42) | <0.0001 | 7278 (29.03) | <0.0001 |
| 60-79 | 73782 (37.63) | 8721 (25.30) |  | 12199 (48.66) |  |
| ≥80 | 48671 (24.82) | 7339 (21.29) |  | 5592 (22.31) |  |
| **Gender** (female) | 97938 (49.95) | 22110 (64.13) | <0.0001 | 9925 (39.59) | <0.0001 |
| **Pre-existing clinical conditions**, n (%)  (in the previous 4 years) | | | | | |
| Ischemic stroke | 1400 (0.71) | 352 (1.02) | <0.0001 | 402 (1.60) | <0.0001 |
| Acute heart failure | 498 (0.25) | 79 (0.23) | 0.3950 | 172 (0.69) | <0.0001 |
| Chronic heart failure | 747 (0.38) | 172 (0.50) | 0.0013 | 231 (0.92) | <0.0001 |
| Myocardial infarction | 2271 (1.16) | 359 (1.04) | 0.0596 | 629 (2.51) | <0.0001 |
| Chronic ischemic heart disease | 4595 (2.34) | 690 (2.00) | <0.0001 | 1400 (5.58) | <0.0001 |
| Coronary artery disease | 547 (0.28) | 77 (0.22) | 0.0668 | 142 (0.57) | <0.0001 |
| Angina | 1652 (0.84) | 202 (0.59) | <0.0001 | 435 (1.74) | <0.0001 |
| Cardiogenic shock | 53 (0.03) | 14 (0.04) | 0.1725 | 45 (0.18) | <0.0001 |
| Atrial fibrillation | 4957 (2.53) | 1013 (2.94) | <0.0001 | 1610 (6.42) | <0.0001 |
| Venous thromboembolism | 1030 (0.53) | 288 (0.84) | <0.0001 | 743 (2.96) | <0.0001 |
| Chronic kidney disease | 1871 (0.95) | 519 (1.51) | <0.0001 | 851 (3.39) | <0.0001 |
| Dialysis | 142 (0.07) | 112 (0.32) | <0.0001 | 159 (0.63) | <0.0001 |
| Chronic obstructive disease | 3611 (1.84) | 1223 (3.55)↑ | <0.0001 | 6224 (24.83)↑ | <0.0001 |
| Asthma | 259 (0.13) | 62 (0.18) | 0.0283 | 154 (0.61) | <0.0001 |
| Liver disease | 1003 (0.51) | 233 (0.68) | 0.0001 | 399 (1.59) | <0.0001 |
| **Charlson index**  (in the previous 12 months)  (ys), mean ± SD | 0.06 ± 0.39 | 0.09 ± 0.54 | <0.0001 | 0.32 ± 1.17 | <0.0001 |
| **CV healthcare utilization markers,** n (%)  (in the previous 12 months) | | | | | |
| ECG | 31617 (16.12) | 4228 (12.26) | 0.0008 | 5590 (22.30) | <0.0001 |
| Cardiology visit | 24863 (12.68) | 2983 (8.65) | <0.0001 | 4511 (17.99) | <0.0001 |
| **Medications,**  n (%) (in 2019) |  |  |  |  |  |
| Antidiabetic drugs | 17808 (9.08) | 2132 (6.18) | <0.0001 | 3932 (15.68) | <0.0001 |
| ACE-I/ARBS | 64995 (33.15) | 7759 (22.51) | <0.0001 | 10703 (42.69) | <0.0001 |
| Beta blockers | 37350 (19.05) | 4796 (13.91) | <0.0001 | 6604 (26.34) | <0.0001 |
| Diuretics | 17726 (9.04) | 2489 (7.22) | <0.0001 | 3755 (14.98) | <0.0001 |
| Ca-antagonists | 22984 (11.72) | 2658 (7.71) | <0.0001 | 4056 (16.18) | <0.0001 |
| Lipid lowering drugs | 40497 (20.65) | 4271 (12.39) | <0.0001 | 6984 (27.86) | <0.0001 |
| Antiplatelet drugs | 25901 (13.21) | 3049 (8.84) | <0.0001 | 4794 (19.12) | <0.0001 |
| Oral anticoagulant drugs | 10989 (5.60) | 1277 (3.70) | <0.0001 | 2119 (8.45) | <0.0001 |
| Heparin | 9286 (4.74) | 1621 (4.70) | 0.7855 | 1774 (7.08) | <0.0001 |
| Drugs for respiratory disease | 18835 (9.61) | 2968 (8.61) | <0.0001 | 3432 (13.69) | <0.0001 |
| Steroidal anti-inflammatory drugs | 15101 (7.70) | 2623 (7.61) | 0.5507 | 2861 (11.41) | <0.0001 |
| No-steroidal anti-inflammatory drugs | 27242 (13.89) | 3978 (11.54) | <0.0001 | 4308 (17.18) | <0.0001 |
| **Abbreviations**: ACE-I, angiotensin-converting enzyme inhibitors; ARBs, angiotensin II receptor agonist blockers; SD, standard deviation. | | | | | |

**Table S2.** Baseline characteristics of COVID-19 hospitalized and non-hospitalized patients and control subjects alive after 30 days, before IPTW, during the 2^nd^/3^rd^ wave of the pandemic.

| **Variables** | **CONTROL**  **N=1316933** | **Non- hospitalized**  **N=384549** | **P-value**  **vs Ctrl** | **Hospitalized**  **N=41051** | **P-value**  **vs Ctrl** |
| --- | --- | --- | --- | --- | --- |
| **Age** (ys)**,** mean ± SD | 60.12 ± 13.87 | 58.06 ± 12.92 | <0.0001 | 70.48 ± 13.40 | <0.0001 |
| **Age groups** (ys), n (%) | | | | | |
| 40-59 | 745308 (56.59) | 239924 (62.39) | <0.0001 | 9724 (23.69) | <0.0001 |
| 60-79 | 408585 (31.03) | 112270 (29.20) |  | 18997 (46.28) |  |
| ≥80 | 163040 (12.38) | 32355 (8.41) |  | 12330 (30.04) |  |
| **Gender** (female) | 681932 (51.78) | 204435 (53.16) | <0.0001 | 17146 (41.77) | <0.0001 |
| **Pre-existing clinical conditions**, n (%)  (in the previous 4 years) | | | | | |
| Ischemic stroke | 4160 (0.32) | 1196 (0.31) | 0.6352 | 682 (1.66) | <0.0001 |
| Acute heart failure | 1313 (0.10) | 370 (0.10) | 0.5453 | 307 (0.75) | <0.0001 |
| Chronic heart failure | 2047 (0.16) | 635 (0.17) | 0.1826 | 470 (1.14) | <0.0001 |
| Myocardial infarction | 8578 (0.65) | 2593 (0.67) | 0.1213 | 1120 (2.73) | <0.0001 |
| Chronic ischemic heart disease | 15241 (1.16) | 4519 (1.18) | 0.3638 | 2275 (5.54) | <0.0001 |
| Coronary artery disease | 2161 (0.16) | 652 (0.17) | 0.4638 | 335 (0.82) | <0.0001 |
| Angina | 6446 (0.49) | 1947 (0.51) | 0.1898 | 744 (1.81) | <0.0001 |
| Cardiogenic shock | 258 (0.02) | 85 (0.02) | 0.3342 | 64 (0.16) | <0.0001 |
| Atrial fibrillation | 14983 (1.14) | 4580 (1.19) | 0.0064 | 2625 (6.39) | <0.0001 |
| Venous thromboembolism | 3542 (0.27) | 1299 (0.34) | <0.0001 | 1010 (2.46) | <0.0001 |
| Chronic kidney disease | 5383 (0.41) | 1739 (0.45) | 0.0002 | 1430 (3.48) | <0.0001 |
| Dialysis | 473 (0.04) | 292 (0.08) | <0.0001 | 193 (0.47) | <0.0001 |
| Chronic obstructive disease | 11215 (0.85) | 4504 (1.17) | <0.0001 | 5564 (13.55)↑ | <0.0001 |
| Asthma | 1074 (0.08) | 392 (0.10) | 0.0002 | 184 (0.45) | <0.0001 |
| Liver disease | 4036 (0.31) | 1239 (0.32) | 0.1228 | 565 (1.38) | <0.0001 |
| **Charlson index**  (in the previous 12 months)  (ys), mean ± SD | 0.03 ± 0.26 | 0.04 ± 0.31 | <0.0001 | 0.28 ± 0.93 | <0.0001 |
| **CV healthcare utilization markers,** n (%)  (in the previous 12 months) | | | | | |
| ECG | 116390 (8.84) | 35840 (9.32) | <0.0001 | 7748 (18.87) | <0.0001 |
| Cardiology visit | 87789 (6.67) | 25482 (6.63) | 0.3848 | 6283 (15.31) | <0.0001 |
| **Medications**, n (%)  (In the previous 12 months) | | | | | |
| Antidiabetic drugs | 80909 (6.14) | 22693 (5.90) | <0.0001 | 7370 (17.95) | <0.0001 |
| ACE-I/ARBS | 305393 (23.19) | 86530 (22.50) | <0.0001 | 18238 (44.43) | <0.0001 |
| Beta blockers | 173023 (13.14) | 47783 (12.43) | <0.0001 | 11820 (28.79) | <0.0001 |
| Diuretics | 68160 (5.18) | 17094 (4.45) | <0.0001 | 7288 (17.75) | <0.0001 |
| Ca-antagonists | 98089 (7.45) | 26188 (6.81) | <0.0001 | 7441 (18.13) | <0.0001 |
| Lipid lowering drugs | 188857 (14.34) | 52064 (13.54) | <0.0001 | 12162 (29.63) | <0.0001 |
| Antiplatelet drugs | 100857 (7.66) | 26027 (6.77) | <0.0001 | 8430 (20.54) | <0.0001 |
| Oral anticoagulant drugs | 42837 (3.25) | 10547 (2.74) | <0.0001 | 4167 (10.15) | <0.0001 |
| Heparin | 41620 (3.16) | 27755 (7.22) | <0.0001 | 6126 (14.92) | <0.0001 |
| Drugs for respiratory disease | 81527 (6.19) | 30104 (7.83) | <0.0001 | 5618 (13.69) | <0.0001 |
| Steroidal anti-inflammatory drugs | 71708 (5.45) | 42859 (11.15) | <0.0001 | 8012 (19.52) | <0.0001 |
| No-steroidal anti-inflammatory drugs | 133312 (10.12) | 46216 (12.02) | <0.0001 | 7192 (17.52) | <0.0001 |
| **Abbreviations:** ACE-I, angiotensin-converting enzyme inhibitors; ARBs, angiotensin II receptor agonist blockers; SD, standard deviation. | | | | | |

**Table S3.** Post-acute frequency and HRs (95%, CI) of Health care markers in COVID-19 group compared to control group during the 1^st^ and 2^nd^/3^rd^ wave of the pandemic.

| **Variables** | **1^st^ wave** | | | **2^nd^/3^rd^ wave** | | |
| --- | --- | --- | --- | --- | --- | --- |
|  | **COVID-19**  N=59,545  N, (%) | **Control**  N=196,091  N, (%) | **HR (95%, CI)** | **COVID-19**  N=425,600  N, (%) | **Control**  N=1,316,933  N, (%) | **HR (95%, CI)** |
| ECG | 9,048  (15.20) | 16,489  (8.41) | 1.91 (1.86, 1.96) | 45,176  (10.61) | 97,939  (7.44) | 1.41 (1.40, 1.43) |
| Cardiology visits | 5,629  (9.45) | 13,304  (6.78) | 1.46 (1.42, 1.51) | 33,351  (7.84) | 74,159  (5.63) | 1.37 (1.35, 1.39) |
| ER access | 10,908 (18.32) | 22,557  (11.50) | 1.69 (1.63, 1.71) | 61,069  (14.35) | 143,994 (10.93) | 1.30 (1.29, 1.32) |
| Re-hospitalization for CV disease | 47  (0.08) | 94  (0.05) | 1.79 (1.26, 2.55) | 191  (0.04) | 477  (0.04) | 1.20 (0.99, 1.42) |

Healthcare markers of interest were ascertained from 30 days of COVID-19 diagnosis until the end of follow-up (index date + 9 months). Re-hospitalization of interest consisted of hospital admission due to ischemic stroke, acute heart failure, chronic heart failure, myocardial infarction, chronic ischemic heart disease, coronary artery disease, angina, cardiogenic shock, atrial fibrillation and venous thromboembolism. Hazard ratios (HRs) were computed after IPTW. ER: emergency room

HRs (95% CI) were adjusted for sex, age, pre-existing condition, medications of interest and previous cardiologists visits and ECG. IPTW 1st wave (mean, median [q1, q3]) = 1.00, 0.98 [0.93, 1.04]. IPTW 2nd/3rd wave (mean, median [q1, q3]) = 1.00, 0.99 (0.96,1.02)

**Table S4.** Negative outcome controls

| **Outcomes** | **1^st^ wave** | | | **2^nd^/3^rd^ wave** | | |
| --- | --- | --- | --- | --- | --- | --- |
|  | **COVID-19**  N=59,545  N, (%) | **Control**  N=196,091  N, (%) | **HR (95%, CI)** | **COVID-19**  N=425,600  N, (%) | **Control**  N=1,316,933  N, (%) | **HR (95%, CI)** |
| Skull fractures | 10 (0.02) | 32 (0.02) | 1.01 (0.49, 2.08) | 60 (0.01) | 146 (0.01) | 1.32 (0.97, 1.78) |
| Malignant melanoma of skin | 3 (0.01) | 7 (0.00) | 1.90 (0.52, 7.02) | 26 (0.01) | 69 (0.01) | 1.22 (0.78, 1.91) |
| Malignant neoplasm of tongue | 1 (0.00) | 9 (0.00) | 0.42 (0.06, 3.16) | 18 (0.00) | 43 (0.00) | 1.22 (0.70, 2.13) |
| Carcinoma in situ of skin | 2 (0.00) | 4 (0.00) | 2.96 (0.63, 14.03) | 7 (0.00) | 14 (0.00) | 1.65 (0.68, 4.01) |
| Hodgkin’s disease | 4 (0.01) | 5 (0.00) | 3.19 (0.88, 11.55) | 11 (0.00) | 18 (0.00) | 1.25 (0.55, 2.85) |
| Composite negative outcome* | 10 (0.02) | 25 (0.01) | 1.76 (0.88, 3.55) | 62 (0.01) | 144 (0.01) | 1.27 (0.94, 1.71) |

Outcomes were ascertained 30 days after the COVID-19 diagnosis until the end of follow-up (up to 9 months after the index date). Composite negative outcome consisted of hospitalization by melanoma of skin + neoplasm of tongue + carcinoma of skin + Hodgkin’s disease. Hazard ratios (HRs) were computed after IPTW. HRs (95% CI) were adjusted for sex, age, pre-existing condition, medications of interest and previous cardiologist visits and ECG. IPTW 1st wave (mean, median [q1, q3]) = 1.00, 0.98 [0.93, 1.04]. IPTW 2nd/3rd wave (mean, median [q1, q3]) = 1.00, 0.99 (0.96,1.02). CI, confidence interval

# **ICD9-CM codes for diagnosis and procedures of interest**

| **International Classification of Disease, Ninth Revision DM (ICD9-CM code) for diagnosis and procedure** | |
| --- | --- |
| **Diagnosis and procedures** | **ICD-9 Code** |
| **Ischemic stroke** | 433.01, 433.11, 433.21, 433.31, 433.81, 433.91, 434.01, 434.91 |
| **Chronic ischemic heart disease** | 414.X |
| **Coronary artery disease** | 411, 411.8, 411.81, 411.89, 36.X |
| **Acute heart failure** | 428.21, 428.31, 428.41 |
| **Chronic heart failure** | 428.23, 428.33, 428.43, 402.01, 402.11, 402.91 |
| **Myocardial infarction** | 410.x |
| **Angina** | 411.1, 413, 413.0, 413.1, 413.9 |
| **Atrial fibrillation** | 427.31, 427.32 |
| **Cardiogenic shock** | 785.51 |
| **Venous thromboembolism** | 325, 415.1, 415.11, 415.19, 451.1, 451.11, 451.19, 451.2, 451.8, 451.81, 451.83, 451.84, 451.89, 451.9, 453.2 453.3, 453.40, 453.41, 453.42, 453.8, 453.9, |
| **Dialysis** | V451, 39.95 |
| **Chronic kidney disease** | 585.X |
| **Chronic obstructive disease** | 491.X , 492.X, 494.X, 496.X, 518.81, 518.83, 518.84 |
| **Asthma** | 493.X |
| **Liver disease** | 571.X, 573.X, 070.X, 570.X, 572.X |
| **Non-osteoporotic fractures** | 800.X, 801.X, 802.X, 803.X, 804.X, 806.X, 807.X, 809.X, 810.X, 811.X, 812.X, 813.X, 814.X, 815.X, 816.X, 817.X, 818.X, 819.X, 822.X, 824.X, 825.X, 826.X, 827.X, 828.X, 829.X |
| **ECG** | 8952, 8950 |
| **Cardiovascular visit** | 89013, 897A3 |

# [**ATC**](https://en.wikipedia.org/wiki/Anatomical_Therapeutic_Chemical_Classification_System) **codes for medications of interest**

| **Drugs** | **ATC-Code** |
| --- | --- |
| **Antidiabetic drugs** |  |
| Insulin | A10A* |
| Metformin | A10BA*; A10BD01; A10BD02; A10BD03; A10BD05; A10BD07  A10BD08; A10BD10; A10BD11; A10BD13; A10BD14; A10BD15;  A10BD16; A10BD17; A10BD18; A10BD20 |
| Sulfonylureas | A10BB*; A10BD01; A10BD02; A10BD04; A10BD06 |
| Glinides | A10BX02; A10BX03; A10BX08; A10BD14 |
| Acarbose | A10BF*; A10BD17 |
| Glitazones | A10BG*; A10BD03; A10BD04; A10BD05; A10BD06; A10BD09; A10BD12 |
| DPP-4i | A10BH*; A10BD07; A10BD08; A10BD09;  A10BD10; A10BD11; A10BD12; A10BD13; A10BD18; A10BD19; A10BD21; A10BH51 |
| GLP-1 RA | A10BX04; A10BX07; A10BX10; A10BX13; A10BX14; A10BJ* |
| SGLT-2i | A10BK*; A10BX09; A10BX11; A10BX12; A10BD15; A10BD16; A10BD19; A10BD20; A10BD21 |
| **ACE-I/ARBS** | C09*; C10BX10 |
| **Beta blockers** | C07* |
| **Diuretics** | C03* |
| **Ca-antagonists** | C08* |
| **Lipid-lowering drugs** | C10* |
| **Antiplatelet drugs** | N02BA01; B01AC*; C10BX08; C10BX02; C10BX05; C10BX01 |
| **Oral anticoagulants** | B01AA03; B01AA07;  B01AE07; B01AF01; B01AF02; B01AF03 |
| **Heparin** | B01AB*; B01AX* |
| **Drugs for respiratory disease** | R03AC12, R03AC13, R03AC18, R03AC19, R03AK06, R03AK07, R03AK08, R03AK10, R03AK11, R03AL03, R03AL04, R03AL05, R03AL06, R03AL09; R03AC02, R03AC03, R03AC04, R03CC02, R03CC04, R03AL01, R03AL02; R03BA01, R03BA05, R03BA03, R03BA02, R03BA08; R03BB01, R03BB02, R03BB04, R03BC01, R03BC03, R03DA04, R03DC01, R03DC03 |
| **Steroidal anti-inflammatory drugs** | H02AB* |
| **Non-steroidal anti-inflammatory drugs** | M01*; N02B* |
| **Abbreviations:** ATC, Anatomical Therapeutic Chemical Classification; DPP-4i, dipeptidyl peptidase-4 inhibitors; GLP-1 RA, Glucagon-like peptide-1 receptor agonists; SGLT-2i, Sodium-glucose co-transporter-2 (SGLT2) inhibitors; ACE-I, angiotensin-converting enzyme inhibitors; ARBs, angiotensin II receptor agonist blockers. | |
